# The Association of Number and Space Under Different Tasks: Insight From a Process Perspective

**DOI:** 10.3389/fpsyg.2018.00957

**Published:** 2018-06-12

**Authors:** Zhijun Deng, Yinghe Chen, Meng Zhang, Yanjun Li, Xiaoshuang Zhu

**Affiliations:** ^1^School of Developmental Psychology, Faculty of Psychology, Beijing Normal University, Beijing, China; ^2^Department of Psychology, Rutgers, The State University of New Jersey, New Brunswick, NJ, United States

**Keywords:** SNARC effect, parity judgment task, magnitude classification task, phase-to-phase design and analyses, process perspective

## Abstract

We investigated the Spatial Numerical Association of Response Codes (SNARC) effect in 240 adults using a parity judgment and a magnitude classification task. Eight numbers from 1 to 9 except 5 were randomly presented one at a time, half of the participants were asked to compare these number with the target number 5 in the magnitude classification task; the other half of the participants were asked to judge whether these numbers were even or odd. It was called a phase when all eight numbers were tested; there were in total 16 phases. Detailed analyses of the changes in response times across the range of numbers and the chronological trend of the SNARC effect size over 16 phases estimated by a curvilinear regression model were reported. This phase-to-phase design and analyses provide an insight into the process of the SNARC effect in different tasks. We found that the SNARC effect emerged earlier and stayed more stable in magnitude classification task than in the parity task during the time course. Furthermore, the size of SNARC effect increased with time in the magnitude classification task, whereas it fluctuated up and down over time in the parity task. These findings indicate that the association of the number and space is dynamic and the process of the SNARC effect varies across tasks.

## Introduction

It is well known that the processing of numerical magnitude is closely related to spatial processing in the domain of numerical cognition (Wood et al., 2008; Fias et al., 2011). The Spatial Numerical Association of Response Codes (SNARC) effect refers to the phenomenon that individuals typically react faster to relatively smaller numbers with left-sided responses and they react faster to relatively larger numbers with right-sided responses. It is one of the most striking demonstrations of the numerical-spatial association (Dehaene et al., 1993). The SNARC effect has long been ascribed to a mental number line stored in the long-term memory (Dehaene, 1992; Gevers et al., 2003).

However, accumulating evidence suggests that many transient factors can affect The SNARC effect. For instance, the given number range and the reference number affect participants’ left or right side responses (Dehaene et al., 1993; Fias et al., 1996; Ben Nathan et al., 2009): The number 5 receives faster right side responses when the overall range is 1–5, but it receives faster left side responses when the range is 4–9 (Dehaene et al., 1993). Moreover, task instructions also affect the SNARC effect (Ristic et al., 2006; Viarouge et al., 2014a): asking participants to imagine a linear rule leads to a standard SNARC effect, whereas asking them to imagine a circular clock leads to a reversed SNARC effect (Bächtold et al., 1998). Additionally, researchers also found that the directional component of a prior spatial activity (e.g., directions in number placement or text-reading) modulated the strength of the SNARC effect (Shaki and Fischer, 2008; Fischer et al., 2010). These findings indicate that spatial–numerical associations are not fixed; they can be affected by tasks and measurements.

An impressive number of studies on spatial-numerical associations using the repetition design, which presented numerous repetitions of single digits (Wood et al., 2008; Fischer and Shaki, 2014), but only a few focused on the repetition effect. For example, in order to explore the exact task factors that affect the SNARC effect, some studies focused more precisely on the trial-to-trial changes (Notebaert et al., 2006; Fischer et al., 2010; Pfister et al., 2013). Pfister et al. (2013) examined the effect of prior trials on the SNARC effect, specifically how the preceding congruency between the target number’s spatial association and the required response influenced the SNARC effect. They asked participants to perform a parity judgment task, and found that the size of SNARC effect was reduced instantly after participants experienced the preceding incongruence. Studies (Notebaert et al., 2006; Fischer et al., 2010; Pfister et al., 2013) with such sequential modulation provide a finer measurement of the dynamics of the SNARC effect, and indicate that the spatial–numerical associations could be a real-time control process. However, this trial-to-trial design may be useful for parity judgment tasks, but it can not be applied to a magnitude classification task, because in a magnitude classification task, congruent trials and incongruent trials are often separated into two blocks. The instant control over spatial-numerical associations like Pfister et al. (2013) can not be obtained in a magnitude classification task.

Both magnitude classification tasks and parity judgment tasks are commonly used methods for investigating the SNARC effect. In parity judgment tasks, participants are asked to judge whether digits are odd or even; in magnitude classification tasks, participants are asked to judge whether digits are smaller or larger than a reference number. Whether these two types of tasks involve the same processes of spatial–numerical associations are controversial.

Some studies found that the number-space associations measured by the parity task and magnitude classification task shared common processes. For example, Cheung et al. (2015) found a significant correlation between the sizes of SNARC effects in these two tasks (see also Georges et al., 2017). Furthermore, several studies suggest that the number-space associations in both parity and magnitude processing tasks arise from the verbal-spatial coding mechanisms (Gevers et al., 2010; Imbo et al., 2012). Though these findings suggest a single predominant account, accumulating evidence has indicated the task-dependent coding mechanism.

The magnitude classification task and the parity judgment task differ in many ways, therefore they may capture different aspects of spatial–numerical associations. First, the processing of magnitude information is different, magnitude information is implicitly and automatically activated in parity judgment task, whereas it is not the case for the magnitude task, in which numerical magnitude is task-relevant and needs to be processed voluntarily (Priftis et al., 2006; Shaki and Fischer, 2018); Second, the difference also exists in the response selection stage, for magnitude classification, the same responses were associated with numbers that were smaller or larger than the referent, whereas for parity judgment, the responses alternate for each number (van Dijck et al., 2009). Besides, parity judgment has a unique effect, i.e., the MARC effect (Nuerk et al., 2004; Roettger and Domahs, 2015), where odd numbers are responded faster on left hand side and even numbers are responded faster on right hand side. This effect is usually not present in the magnitude classification task. Furthermore, studies using both tasks showed that the number-space mapping required different modalities (Herrera et al., 2008; van Dijck et al., 2009) and different amounts of working memory resources (Deng et al., 2017) for magnitude classification and parity judgment. Thus, it is desirable to explore the differences of number-space association process that is involved in magnitude classification tasks and parity judgment tasks.

The present study examined the differences in the number-space association process using the magnitude classification task and the parity judgment task. Unlike previous researchers focusing on the influence of a prior trial on the next trial (as in Notebaert et al., 2006; Fischer et al., 2010; Pfister et al., 2013), we think the process of state changes trial-to-trial, throughout all the trials (Macdonald et al., 2011; Unsworth and McMillan, 2014; Amir et al., 2016). Therefore, we adopted the trial-to-trial processing perspective and investigated the phase-to-phase changes for each participant. In our study, eight numbers from 1 to 9 (except 5) were tested in random order for both tasks; the unit of eight trials was considered as a phase. Our study was designed and data analyses (e.g., regression) were conducted in a phase-to-phase manner. We report changes of the SNARC effect during the time course of all 16 phases, where the size of the SNARC effect was represented by the regression coefficients. Our phase-to-phase design and analyses provided a micro-level perspective for better understanding the process of number and space association and its variations in different numerical tasks.

## Materials and Methods

### Participants

A total of 240 native Chinese adults participated the experiment. All were right-handed and reported normal or corrected-to-normal vision. We divided participants randomly into two groups each with 120 adults: one group (74 females, 46 males; 18–28 years old, mean age 22.68 years) was assigned to complete the magnitude classification task, and the other group (73 females, 47 males; 18–29 years old, mean age 22.32 years) was assigned to complete the parity judgment task. None of the participants were familiar with the purpose of the study. We explained the procedures of the experiment and obtained participants’ informed consent before experiment. Participant each received a small amount of monetary reward after experiment.

### Stimuli and Procedure

The experiment was programmed using E-Prime 2 Professional Software on a 17-in. LCD computer screen (1,280 × 1,024 pixels). Stimuli were Arabic numbers (Arial font, 48 point size) in the range from 1 to 9 with the exception of 5. Between stimulus presentations, participants saw a fixation point, which was an asterisk (^∗^) of size 48 points in the center of the screen. All stimuli were in black showing on a white background. Participants indicated their judgments by pressing either the A or L key on a standard QWERTY computer keyboard.

For both the magnitude classification task and parity judgment task, each trial started with a 300 ms presentation of the fixation asterisk, then a target number appeared in the center of the screen. Participants had to make their judgments within 5000 ms by pressing corresponding keys. In magnitude classification task, participants were asked to judge whether digits are smaller or larger than a reference number. In parity judgment task, participants were asked judge whether digits are odd or even. There would be a 1000 ms of blank screen following each trial. Participants’ response accuracy and response time were recorded.

For each task, we presented a total of 128 trials (8 numbers × 16 phases) in two blocks. Each block contained 8 successive phases. In each phase, all of the 8 numbers (i.e., 1, 2, 3, 4, 6, 7, 8, and 9) were tested in a random order. These two blocks differed in their response mapping. In magnitude classification task, we had one block that mapped small numbers on the left side and large numbers on the right side, and the other block that counterbalance the mapping. In parity judgment task, we had one block that associated even numbers with left side and odd numbers with right side, and the other block that counterbalance the association. The order of blocks was also counterbalance across participants. Before testing, participants completed six practice trials to become familiar with the procedure. Phases were labeled in the order of their occurrence, continuously numbered from the first phase of the first block to the last phase of the second block.

## Results

### Data Treatment

We excluded trials with errors for data analyses. There were 2.29% of trials were with error for magnitude classification task; there were 3.82% of trials were with error for parity judgment task. Additionally, when participants’ RT deviated from the corresponding cell mean by more than 3 standard deviations, we considered this data as outliers. There were 0.91% outliers in magnitude classification task and 1.26% outliers in parity judgment task.

### Response Times

**FIGURE 1:**
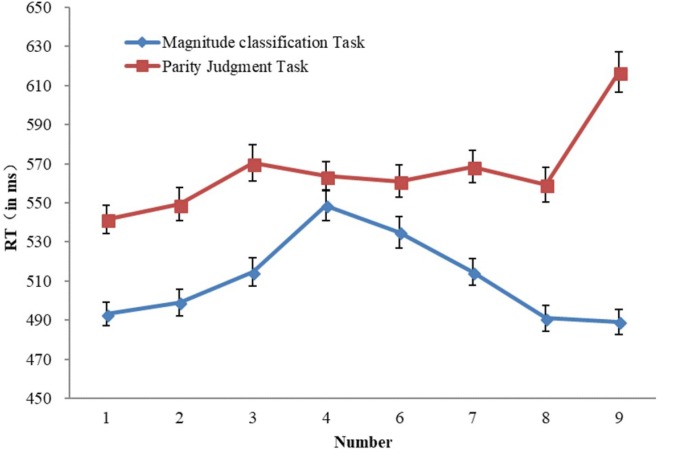
Mean response time (RT) for magnitude classification task and parity judgment task as a function of number magnitudes.

The mean RTs and standard error of the mean (SEM) of responses to each number magnitude in magnitude classification task and parity judgment task were calculated (See **Figure 1**). We performed a 2 (type of task: magnitude classification task, parity judgment task) × 8 (number magnitude: 1, 2, 3, 4, 6, 7, 8, and 9) repeated measures ANOVA on mean reaction times. The results revealed significant main effects of Task, *F*(1,238) = 29.20, *p* < 0.0001, η^2^ = 0.109, and Number magnitude, *F*(7,1666) = 46.30, *p* < 0.0001, η^2^ = 0.163. The interaction was also significant, *F*(7,1666) = 63.02, *p* < 0.0001, η^2^ = 0.209. Further, one way of simple effect analyses indicated that, the RTs for all the numbers in parity judgment task were significantly longer than those in magnitude classification task (all *ps* < 0.05) except for the digit 4 (*p* = 0.165). The other way of simple effects analyses suggested that, in the magnitude classification task, RTs for both 4 and 6 were significantly longer than those for any other digit (all *ps* < 0.001); RTs for 1, 2, 8, and 9 were significantly shorter than those for 3, 4, 6, and 7 (all *ps* < 0.05). In the parity judgment task, the RT for 1 was significantly shorter than those for others (all *ps* < 0.001); the RT for 9 was significantly longer than those for others (all *ps* < 0.0001).

In general, RTs for the parity judgment task were longer than those for the magnitude classification task. RTs changed across the eight number magnitudes differently in magnitude classification task and parity judgment task. In magnitude classification task, RT reflected a distance effect (Moyer and Landauer, 1967; Sekuler and Mierkiewicz, 1977), which means that RTs decreased when the distance between the standard and the target increased. In the parity judgment task, RT reflected a size effect (Starkey and Cooper, 1980; Dehaene et al., 1998), which means that RTs increased as the magnitude increases.

### SNARC Effects at Individual Level and at Group Level

The SNARC effect traditionally has been indicated by the existence of a difference of response time to the same number between using left hand and using right hand, which oftentimes favors the right hand for numbers greater than 5 and the left hand for numbers less than 5.

For each participant and each number magnitude, we calculated an RT difference (dRT) for each participant by subtracting the mean RT using the left hand from the mean RT using the right hand and regressed the dRT on number magnitudes (i.e., 1–4, 6–9). The regression weights of each participant indicated their SNARC effect (Fias et al., 1996), which were used for further analyses. For both magnitude classification and parity judgment task, we examined whether the regression weights deviated significantly from zero at the group by using *t*-tests. For the magnitude classification task, *M* = -6.25, *SD* = 17.84, *t*(119) = -3.836, *p <*0.0001. For the parity judgment task, *M* = -7.59, *SD* = 9.92, *t*(119) = -8.39, *p <*0.0001. In both tasks, the slopes were significantly different from zero, indicating the presence of the SNARC effect at the group level. Moreover, an independent samples *t*-test was applied to compare the regression weights for magnitude classification task and parity judgment task. We found the difference was not significant, *t*(238) = 0.72, *p* = 0.47. The sizes of the SNARC effect in these two tasks were equal at the group level.

Additionally, as expected, the majority of participants showed the SNARC effect, which was negatively associated with the number magnitude. There were 69.2% of participants (i.e., 83) in the magnitude classification task and 75.8% of participants (i.e., 91) in the parity judgment task showed such effect.

### SNARC Effects Across Phases

The mean RTs and standard deviations of each phase for the parity and magnitude tasks were calculated (see **Tables 1, 2**). For the magnitude classification task, we performed repeated measures ANOVA with phase on mean reaction times. The results revealed a significant main effect of phases, *F*(15,1785) = 5.594, *p* < 0.0001, η^2^ = 0.045. The *post hoc* test found that RTs for phase 1 and phase 9 were significantly longer than those for others (all *ps* < 0.05); RTs for the rest of phases did not differ from each other (all *ps* > 0.05). For the parity task, the results of repeated measures ANOVA revealed a significant main effect of phases, *F*(15,1785) = 4.526, *p* < 0.0001, η^2^ = 0.037, the *post hoc* test found that the RT for phase 1 was significantly longer than those for others (*p* < 0.05); RTs for the rest of phases did not differ from each other (all *ps* > 0.05).

**Table 1:** The Means and Standard Deviations of RTs of each phase in magnitude classification task.

	Phase number															
	1	2	3	4	5	6	7	8	9	10	11	12	13	14	15	16
*M*	542.34	516.31	510.39	503.10	497.07	508.76	507.77	500.43	528.23	510.94	500.61	500.65	505.24	508.93	501.73	512.33
*SD*	145.25	127.19	125.77	123.68	110.40	132.77	120.85	123.23	148.25	132.85	125.68	114.09	121.99	124.01	122.19	133.79


**Table 2:** The Means and Standard Deviations of RTs of each phase in parity judgment task.

	Phase number															
	1	2	3	4	5	6	7	8	9	10	11	12	13	14	15	16
*M*	605.61	575.82	560.86	565.73	559.32	558.01	559.90	563.64	573.58	552.17	568.85	564.68	564.04	567.74	546.65	560.74
*SD*	184.30	167.23	153.78	151.15	150.37	156.31	152.01	150.27	161.94	145.05	168.89	156.80	166.15	166.99	154.01	162.13


The SNARC effect was examined for each phase by applying regression analyses on dRT with numerical magnitudes as the predictor. For each phase, we calculated the dRT of each number by subtracting the group-level mean RT using the left hand from the group-level mean RT using the right hand. We were able to calculate dRT this way because for each phase the order of blocks was counterbalanced between participants. There were 60 participants who responded to the number with the right hand and there were other 60 participants who responded to the same number with the left hand. For all 16 phases, we calculated the group-level regression slopes as precise quantifications of the SNARC effect and R^2^ as an indicator of proportion of variance explained by each regression model (Pfister et al., 2013).

As shown in **Tables 3, 4**, all 16 phases showed negative SNARC slopes for both magnitude classification task and parity judgment task. Eleven of the 16 phases in the magnitude classification task showed significantly negative regression slops; two of 16 phases in the parity judgment task showed significantly negative regression slops. R^2^ values across all phases were comparatively low, with most less than 0.4.

**Table 3:** Summary of the regression analysis for dRT in magnitude classification task with phase as a predictor.

Regression statistic	Phase number															
	1	2	3	4	5	6	7	8	9	10	11	12	13	14	15	16
B	-2.04	-4.09	-4.35	-4.96	-5.17	-4.93	-6.65	-6.05	-6.27	-7.68	-7.18	-6.63	-8.57	-10.12	-10.74	-11.38
P	0.54	0.06	0.12	0.04	0.03	0.01	0.01	0.02	0.19	0.02	0.20	0.01	0.01	0.00	0.00	0.05
R^2^	0.07	0.46	0.35	0.53	0.60	0.71	0.73	0.62	0.27	0.64	0.25	0.68	0.70	0.77	0.83	0.51


**Table 4:** Summary of the regression analysis for dRT in parity judgment task with phase as a predictor.

Regression statistic	Phase number															
	1	2	3	4	5	6	7	8	9	10	11	12	13	14	15	16
B	-13.53	-8.57	-5.58	-11.49	-8.20	-4.95	-13.26	-0.20	-1.99	-1.36	-13.78	-2.23	-7.34	-8.22	-16.67	-4.68
p	0.14	0.26	0.45	0.05	0.09	0.29	0.07	0.97	0.83	0.86	0.23	0.75	0.31	0.27	0.01	0.38
R^2^	0.32	0.21	0.10	0.51	0.40	0.19	0.44	0.00	0.01	0.01	0.23	0.02	0.17	0.20	0.70	0.13


For each task, we examined the chronological trend across all 16 phases by applying curve estimation, with time as an independent variable and the regression slops of each phase as the dependent variable.

For the magnitude classification task, the size of the regression slops increased with time, *p* < 0.001; 90.7% of variance were explained by the curvilinear regression model. For the parity judgment task, the chronological trend was not clear (*p* > 0.05) and the model only explained 0.9% of the variances (see **Table 5**).

**Table 5:** Model summary and parameter estimates.

Equation	Model summary				Parameter estimates		
	R Square	F	df1	df2	Sig.	Constant	b1
Magnitude classification	0.907	136.724	1	14	0.000	-2.335	-0.511
Parity judgment	0.009	0.124	1	14	0.730	-8.470	0.099


**FIGURE 2:**
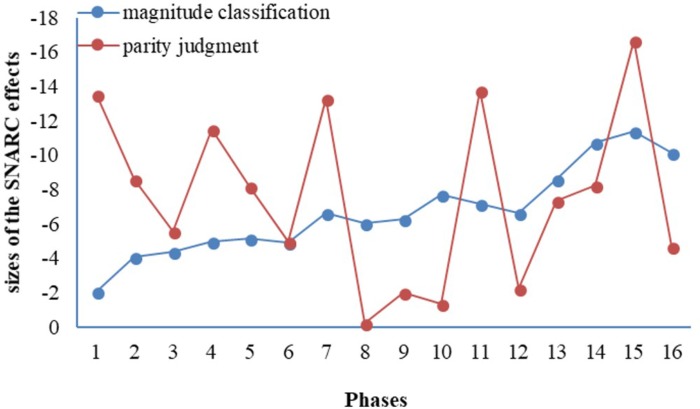
The SNARC effects in the magnitude classification task and parity judgment task as a function of phase.

As show in **Figure 2**, there was a growing trend of SNARC effect throughout the phases in the magnitude classification task, whereas the SNARC effect fluctuated throughout all the phases in the parity judgment task.

## Discussion

We investigated the SNARC effect in a parity judgment and a magnitude classification task with a relatively large sample of participants. Detailed analyses of spatial–numerical associations were reported from the perspective of processes. We observed robust SNARC effects in both the magnitude classification task and the parity judgment task, and most of participants showed negative SNARC effects (Wood et al., 2006a,b). However, analyses on RTs differences among number magnitudes and the phase-to-phase changes revealed different processes for these two tasks. These findings confirmed that the SNARC effect can be easily affected by tasks by providing evidence from the number’s spatial association process.

Though both magnitude classification task and parity judgment task are widely used for exploring the SNARC effect, only a few studies focused on the repetition effect. Previous researchers (Fias et al., 1996; Viarouge et al., 2014b) found that the SNARC effect was relatively stable over sessions or blocks. In our study, we looked into more refined differences between phases within a block. With more detailed analyses regarding the time course, we provided the first evidence for chronological changes of the SNARC effect. The size of SNARC effects increased with time in the magnitude classification task, whereas in the parity task, the values of SNARC effect fluctuated up and down over time. As suggested by Pfister et al. (2013) trial-to-trial design, it is plausible when researchers zoom into the process and conduct more detailed analyses, the refined differences over the process can be observed; hence providing more information about the underlying mechanism of spatial–numerical associations. Moreover, as a repetition design our study was able to detect the temporal differences of SNARC effect also because our analyses of phases were group level and our sample size and number of repetitions provided enough statistical power (Cipora and Nuerk, 2013; Cipora and Wood, 2017).

Furthermore, our results also showed that the SNARC effect in magnitude classification task emerged earlier and stayed more stable than it did in the parity judgment task. In the magnitude classification task, most of the sizes of SNARC effect were significantly negative and increased with time. However, in the parity task, only a few SNARC effects were significantly negative and the values fluctuated up and down over time. This task difference may be because for parity judgment task, a single phase was not long enough to establish a stable association between number and space, making the SNARC effect hard to detect. Also there was a notable dissociation between the RTs of number judgments and the values of the SNARC effect, indicating a different process in making number judgments.

The question that we may ask is why parity judgment and magnitude classification engage different processes over time. One explanation relates to differences between these two tasks, which cashed out the SNARC effect (Georges et al., 2014, 2015). In the current study, the magnitude classification task required participants to process magnitudes; they were also primed by a mental number line, especially when asked to respond to small numbers with left hand and to respond to large numbers with right hand. Whereas in the parity judgment task, the response to the task (judge whether odd or even) influences the presentation of the number-space association, therefore the number-space association for each phase was weak and unstable. The stability difference between the two tasks could also explain why more working memory resources were apparently needed in the parity task than in the magnitude task (Deng et al., 2017). Working memory resources were needed to rule out the influence of the task set in the parity task, whereas they were only needed to account for the inconsistency in the magnitude task.

Overall, the process difference between the parity task and the magnitude task further illustrated that spatial–numerical interactions in implicit and explicit magnitude processing tasks potentially arise from qualitatively different cognitive mechanisms. Some studies indicated the mechanisms difference from a perspective of element analysis. For example, Georges et al. (2017) found the spatial–numerical associations (SNAs) measured by the parity task and the magnitude task correlated with individual’s arithmetic performance, spatial visualization ability and visualization profile differently. van Dijck et al. (2012) found similar parity SNARC effects in normal population and patients but different magnitude SNARC effects between the two populations, indicating different origins for the two SNARC effects. Similarly, their principle analyses also extracted separate components for parity task and magnitude task, suggesting different cognitive processes were engaged. Our study showed the process where spatial–numerical associations varied in implicit and explicit magnitude processing tasks. Besides, participants’ RTs in parity judgment task increased as number magnitudes increased, which corresponded to the size effect (Dehaene et al., 1998). However, their RTs in the magnitude task behaved more categorically – their pattern can be approximated by two parallel horizontal lines – one for numbers smaller than the criterion and one for numbers larger than the criterion. All these results are consistent with Gevers et al. (2006) study, thereby our study further supports the task-dependent spatial coding mechanisms (see also Wood et al., 2008).

A question that cannot be answered based on the present results is whether the differences of the SNARC effect between these two tasks reflect different number-space associations or just different task demands. Previous research pointed out that the SNARC effect was range-dependent (Dehaene et al., 1993), reference-dependent (Bächtold et al., 1998; Ristic et al., 2006), and task demand-dependent (Fischer et al., 2010; Pfister et al., 2013). These characteristics can be considered as evidence for the role of working memory in transient associations of space and number (Fias et al., 2011; De Belder et al., 2015). Alternatively, researcher (Gevers et al., 2006) adopting computational modeling argued for a parallel activation of preexisting links between magnitude and spatial representation and short-term links created on the basis of task instructions. Recent research (Ginsburg and Gevers, 2015; Huber et al., 2016) found that the SNARC effect and the ordinal position effect resulted from the activation of different representations, which supports the computational view of number-space associations.

In conclusion, the present results trace out the process of the number and space association in a magnitude classification task and a parity judgment task. The analyses on RTs differences and the phase-to-phase changes revealed that the formation of the SNARC effect under tasks were different. These findings remind us that the type of task is also a key element in the exploration of the nature of the SNARC effect. More attention and more research need to be done to better understand the nature of SNARC effect and its variations in different tasks. To address the above questions both more empirical evidence and computational models will be helpful in the future.

## Ethics Statement

This research was approved by the local ethical committee of Beijing Normal University. We obtained informed written consent from every participant, according to the institutional guidelines of Beijing Normal University.

## Author Contributions

ZD contributed on designing and conducting the research, on acquisition and interpretation of data, and on drafting of manuscript. YC contributed to conception and design, and on interpretation of data. MZ contributed to conception and design and drafting of manuscript. YL and XZ contributed on interpretation of data. All authors approved the final version of the manuscript for submission.

## Conflict of Interest Statement

The authors declare that the research was conducted in the absence of any commercial or financial relationships that could be construed as a potential conflict of interest.
